# Barcoding (*COI*) Sea Cucumber *Holothuria mammata* Distribution Analysis: Adriatic Rare or Common Species?

**DOI:** 10.3390/genes14112059

**Published:** 2023-11-09

**Authors:** Maya Sertić Kovačević, Ana Baričević, Petar Kružić, Maja Maurić Maljković, Bojan Hamer

**Affiliations:** 1 Laboratory for Marine Biology, Department of Zoology, Faculty of Science, University of Zagreb, Rooseveltov trg 6, 10000 Zagreb, Croatia; maya.sertic@gmail.com (M.S.K.); petar.kruzic@biol.pmf.hr (P.K.); 2 Laboratory for Marine Nanotechnology and Biotechnology, Center for Marine Research, Ruđer Bošković Institute, Giordano Paliaga 5, 52210 Rovinj, Croatia; 3 Laboratory for Evolutionary Ecology, Center for Marine Research, Ruđer Bošković Institute, Giordano Paliaga 5, 52210 Rovinj, Croatia; ana.baricevic@irb.hr; 4 Department of Animal Breeding and Livestock Production, Faculty of Veterinary Medicine, University of Zagreb, Heinzelova 55, 10000 Zagreb, Croatia

**Keywords:** Echinodermata, *Holothuria mammata*, *Holothuria tubulosa*, Adriatic Sea, barcoding, mtDNA, cytochrome *c* oxidase subunit

## Abstract

The overexploitation of the western Pacific Ocean has expanded the sea cucumber fishery into new regions to supply the Asian market. In 2013, sea cucumbers were removed from the Croatian marine protected species list, and commercial fishery took place for a short period (2017–2018) in the Eastern Adriatic Sea. However, holothuroid species are difficult to distinguish. *Holothuria mammata* is a species that has rarely been reported in this region and strongly resembles the common species *Holothuria tubulosa*. This is the first study to assess the genetic diversity of sea cucumbers in the Adriatic Sea using genetic barcoding of the mitochondrial gene cytochrome *c* oxidase subunit 1 (*COI*). Specimens for barcoding were collected from the northern and central Adriatic, along with a specimen that had been previously identified as *H.* sp. cf. *mammata* based on its morphological characteristics. While genetic analyses showed identified this specimen as *H. tubulosa*, 30% of the collected specimens were genetically identified as *H. mammata*. These results call into question the historically accepted sea cucumber assemblage in the Adriatic Sea, which regarded *H. mammata* as a rare species and generally disregarded its presence in large census studies. Such species distribution data are extremely important in developing and monitoring a sustainable fishery.

## 1. Introduction

Sea cucumbers are a traditional delicacy in many Asian countries, including Singapore, Korea, Taiwan, and Hong Kong [1]. Due to overharvesting and stock depletion of these organisms in most of the world’s traditional fisheries, there has been a recent expansion of the industry into temperate regions where sea cucumbers have little economic value in local cultures [2,3,4]. The Mediterranean Sea is among the least harvested regions of the world, with Turkey leading the industry with over 500 tons harvested per year [5,6].

Commercial interest in Adriatic Sea cucumbers can be traced back to the early 1900s, with particular interest placed on the species *Holothuria tubulosa* [7]. More recently, sea cucumbers were commercially exploited in the central Adriatic. The fishery opened in 1994 off the coast of Makarska, and the area was entirely depleted of sea cucumbers by 1995 [8]. The sudden drop in population density compelled the Croatian government to place all sea cucumber species under special protection, prohibiting any form of collection for commercial purposes [9]. This drastic measure was implemented because these organisms’ life history and ecology were relatively unknown, including which species experienced a population decline and why.

Since then, the knowledge gap has not been filled, and the lack of commercial interest in these organisms has also led to a decrease in scientific interest. However, special protection for sea cucumbers was lifted in 2013. After this, a short trial of intensive fishing began, just for them to be protected again from March 2018 until today [10].

Taxonomic research on sea cucumbers in the Adriatic is sparse compared to the Mediterranean Sea, and existing studies have all relied on morphological traits for classification [11,12,13,14,15]. *Holothuria tubulosa* is regarded as the most common species in the region, but the lack of genetic information and population analyses has resulted in uncertainty on the presence of certain species in the Adriatic Sea [4,12,15].

One uncertainty concerns the existence and distribution of *Holothuria mammata* in the Adriatic. While it is included in official species lists of Adriatic Sea cucumbers [9], the species is almost never recorded in scientific literature from the region. Only two individuals have been recorded and described in detail to date, with the specimens (fixed in ethanol) residing in Brussels, Belgium, and Rovinj, Croatia [12]. Zavodnik [12] suggested that *H. mammata* is rare in the Adriatic Sea. However, he was not confident in identifying the two specimens and emphasised the importance of re-evaluating the holothurian species assemblage in the region. Based on Zavodnik’s morphological description of the two *H.* sp. cf. *mammata* specimens, ref. [16] have expressed suspicion that these specimens were misidentified and are *H. tubulosa.* Hence, it has remained unclear whether *H. mammata* inhabits the Adriatic Sea.

Cryptic species are common among the Holothuroidea [17,18], making species identification based on morphological characteristics problematic [4,19]. Morphologically, distinguishing *H. mammata* from *H. tubulosa* is very difficult. Both exhibit high morphological diversity, and many morphotypes overlap between the two species, including ossicle shape, which is often used for sea cucumber species identification [6,16]. Historically, *H. mammata* was distinguished by large mammilate papillae arranged longitudinally on the bivium [20]. However, recent studies have shown that both species can have mammilate and smooth morphotypes [16].

The presence of Cuvierian tubules in *H. mammata* is the most straightforward characteristic distinguishing it from *H. tubulosa*, but these Cuvierian tubules are few in number, small, and never expelled [16,21]. In a pilot study, several *H.* sp. cf. *mammata* specimens, classified as such by their extremely mammilate appearance [20], were dissected to verify their species by the presence of Cuvierian organs. However, no Cuvierian organs were found in any of the examined specimens, suggesting they are *H. tubulosa*. The problem is that the organism needs to be dissected to verify the presence of Cuvierian tubules, which is an ineffective method for simple species identification.

Genetic barcoding has become widely used for taxonomic discrimination of cryptic species. The mitochondrial gene cytochrome *c* oxidase I (*COI*) has been found to accurately classify species in the genus *Holothuria* [19,22], and many mitochondria have already been sequenced [19,23,24,25]. While many specimens have been barcoded in the western and eastern Mediterranean Sea (mainly in the Alboran and Aegean Seas) [26,27,28,29,30], no such efforts and genetic identification have been used to date, for sea cucumber specimens in the Adriatic.

The Adriatic, as the northernmost offshoot of the Mediterranean Sea, is known for having different species distributions compared to other regions of the Mediterranean. The Adriatic Sea is noted as one of the major barriers to larval dispersal in the Mediterranean due to its hydro-geographic isolation, and varying hydrographic conditions in the Ionian Sea heavily impact the species assemblage in this region [27,31]. According to [27], the lack of information on the Adriatic Sea cucumber species distribution has led to a knowledge gap in the gene flow of these Mediterranean Sea organisms.

Our study is a follow-up to previous research on sea cucumbers [11,12,13,14,15]. It aims to clarify the uncertainty considering the presence of *H. mammata* in the Adriatic Sea by using genetic barcoding of the *COI* mitochondrial gene.

## 2. Materials and Methods

Samples: Ten sea cucumbers of the *H.* sp. cf. *tubulosa* morphospecies were collected from 6 locations along the northern and central coast of Croatia between June and August 2015. These locations included Lim Bay (LB), Rovinj (RO), Ičići (IC), Nerezine on the island Cres (CN), Novi Vinodolski (NV), and Biograd na moru (BI) (Table 1). Most samples were collected at depths up to 10 m using skin diving or SCUBA, while samples from offshore Rovinj (RO) were collected using grab sampling at 30 m. Each collection site’s depth and bottom type were noted (Table 1). All sea cucumbers were transported alive in coolers with seawater to the Ruđer Bošković Institute Center for Marine Research (Rovinj, Croatia) on the same day they were collected.

Along with the *H. tubulosa* morphospecies, representative specimens of *H. forskali* and *H. poli* were collected for genetic analysis (barcoding) to check for the correct species assessment. *Holothuria forskali* was easily recognised by the Cuvierian tubules it released when handled, and *H. poli* was recognised by its smaller size, smooth texture, and white-tipped ventral podia [20,32]. All collected specimens were photographed dorsally, ventrally, and laterally.

**Figure 1:**
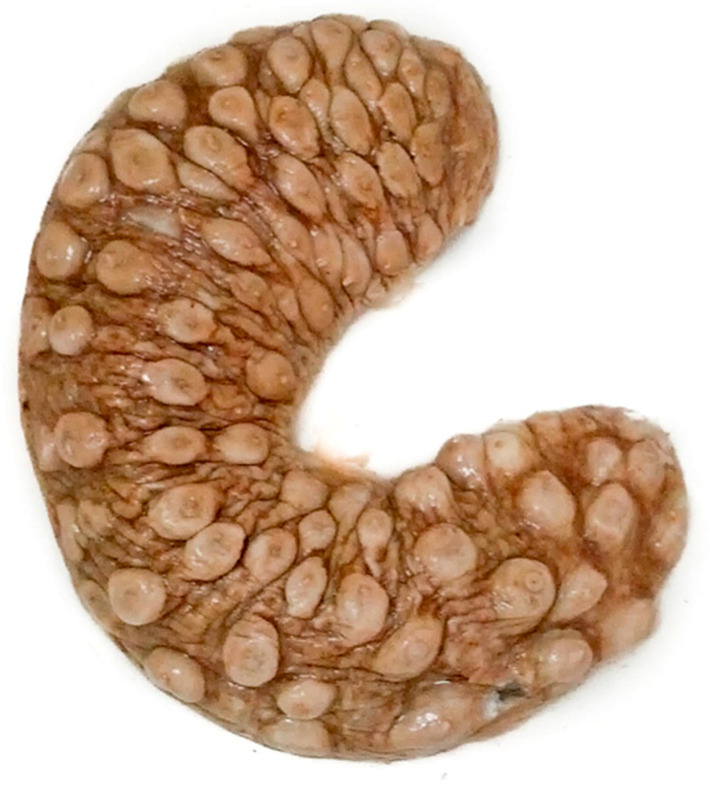
Specimen collected in 1997 and identified as *Holothuria* sp. cf. *mammata*, based on exterior morphology. The specimen is stored in the Ruđer Bošković Institute collection (Cat. No. CMRR 2290) [12]. The specimen’s light colouration is likely due to long-term storage in ethanol. In this study, the specimen is referred to as Z Mljet (ZM1).

Genetic analysis: Six *H.* sp. cf. *tubulosa* individuals from each location (7 individuals from RO) were selected for genetic analysis. Samples of longitudinal muscle tissue were taken from each individual and frozen at −20 °C. The sea cucumbers were kept in tanks with fresh running seawater to allow their wounds to heal before they were returned to the sea. A piece of longitudinal muscle tissue was also removed from the *H.* sp. cf. *mammata* specimen stored in the Ruđer Bošković Institute collection (Figure 1; Cat. No. CMRR 2290) [12].

Genomic DNA was isolated using the DNeasy Blood and Tissue Kit (Qiagen, cat. no. 69504). DNA concentration and purity were estimated using a Nanodrop spectrophotometer (NanoPhotometer, Implen, München, Germany). Based on these values, genomic DNA was diluted from 1:50 to 1:500. According to previous studies on these species in the Mediterranean [27,29], a fragment of the mitochondrial gene cytochrome *c* oxidase I (*COI*) was amplified using PCR and the following primers: COIceF 5′-ACTGCCCACGCCCTAGTAATGATATTT-3′ and COIceR 5′-TCGTGTGTCTACGTCCATTCCTACTGT-3′ [33]. Thermocycling was conducted in a C1000 thermocycler (Bio-Rad T100 PCR Thermal Cycler, Bio-Rad Laboratories, Hercules, CA, USA) with the following conditions: initial denaturation at 95 °C for 4 min, 36 cycles of denaturation at 95 °C for 30 s, annealing at 50 °C for 30 s, extension at 72 °C for 1 min, and ending with a 7 min final extension time at 72 °C. PCR products were purified using a MinElute PCR Purification Kit (Qiagen. cat. no. 28004, Düsseldorf, Germany) according to the manufacturer’s protocol and were sent to Macrogen (Amsterdam, The Netherlands) for sequencing. Each DNA sample was sequenced using both the forward and reverse primers.

Bioinformatics: The raw forward and reverse genetic sequences were assembled, and the resulting consensus sequences were aligned and edited using Geneious Prime ver. 2023.2.1 (Biomatters, Inc., Auckland, New Zealand). The low-quality sections at the ends of each sequence were removed, and the final sequences were cut to the shortest sequence length so they could be compared. Average pairwise tree distances within and between groups were calculated using the Geneious Species Delimitation plugin.

The NCBI GenBank’s Basic Local Alignment Search Tool (BLAST) was used to find corresponding sequences for phylogenetic analysis. All sequences with a 100% cover rate were downloaded, aligned using ClustaW in Mega7 [34] together with our 40 sequences, and cut to the same length. Additionally, the only three available sequences with a 100% cover rate were downloaded for *H. forskali*. After the alignment of all sequences, identification of the different haplotypes was done using DNAsp v6 software [35]. Haplotypes represented by just one sequence retained their name (species, country from where they came, and ordinal number), while haplotypes represented by more than one sequence were grouped under a haplotype name (Table 2). The final alignment comprised of unique haplotypes. Estimation of the most probable nucleotide substitution model for the Neighbor-joining tree using the maximum likelihood method was carried out using the model selection analysis implemented in Mega7 [34]. The model with the lowest BIC score (Bayesian Information Criterion) was considered to best describe the substitution pattern. A maximum likelihood phylogenetic analysis was conducted using the HKY + G + I model [36] with 1000 bootstrap replicates, and the tree with the highest log likelihood is shown. The numbers at nodes indicate bootstrap percentages. The initial tree for the heuristic search was automatically obtained by applying Neighbor-Join and BioNJ algorithms to a matrix of pairwise distances estimated using the Maximum Composite Likelihood (MCL) approach, and the topology with the superior log likelihood value was selected. Within and between mean group/species p-distances were calculated in Mega7 [34].

## 3. Results

This first genetic study of *Holothuria* species in the Croatian Adriatic Sea has resulted in the addition of 40 new *COI* sequences to the NCBI GenBank database, under accession numbers KY774322-KY774361 [37]. In total, 412 *COI* gene base pairs from 196 sequences were analysed: our 40 sequences representing three expected morphospecies (*H. tubulosa*, *H. poli*, and *H. forskali*) and 156 sequences obtained from the NCBI GenBank database. Z Mljet (ZM1; ac. no. KY774360) is a specimen that was extracted from the IRB collection, and was previously described by Zavodnik as *H.* sp. cf. *mammata* [12]. H.f. Rovinj (H. f. RO1; KY774322) and H.p. MDraga (MD1; KY774323) are collected specimens of *H. forskali* and *H. poli* from the Adriatic Sea. After the alignment of all 196 sequences and the identification of different haplotypes, the final alignment comprises 129 haplotypes (see Table 2 for haplotypes represented by more than one sequence and grouped under haplotype names, and Table S1 for complete information on sequences used and identified haplotypes).

**Figure 2:**
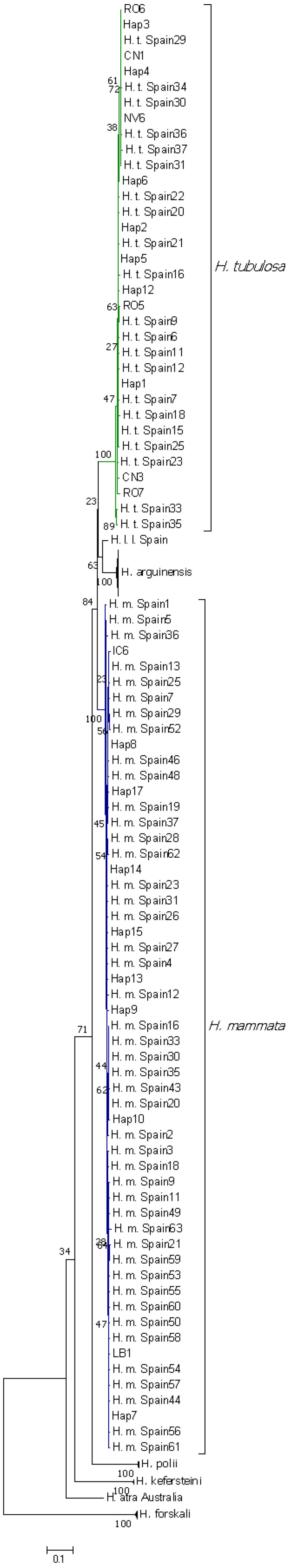
Phylogenetic tree (maximum likelihood), unrooted, based on partial sequences of the *COI* gene obtained from this study and the NCBI GenBank. The analysed specimens from the *Holothuria tubulosa* morphospecies group divide into two distinct groups/species. NCBI GenBank accession numbers for all the sequences used are available in Table S1. The nodes for species other than *H. tubulosa* and *H. mammata* were collapsed for a clearer view of *H. tubulosa*’s and *H. mammata*’s distribution. The full tree, without collapsed nodes, is presented in Figure S1. Collapsed nodes are as follows: *H. arguinensis* Spain 1, 3, 4, 7–9, 11, 12, 14–16, 19–21, 23–33, Hap15, Hap18; *H. polii* Spain 1, *H. polii* Mexico, *H. polii* Italy, Hap11; *H. kefersteini* Mexico 1, 5, Hap19; *H. forskali* Spain 1, 2, *H. forskali* Germany, *H. forskali* RO1.

A comparison of the 38 sequences belonging to the *H.* sp. cf. *tubulosa* morphospecies using the Maximum Likelihood method based on the Hasegawa-Kishino-Yano model with 1000 replicates showed a clear division into two groups/species (Figure 2 and Figure S1). The bootstrap percentage at the node diverging between *H. tubulosa* and *H. mammata* was 84%. The within group/species p-distances calculated for *H. tubulosa* (N = 34) was 0.012, and for *H. mammata* (N = 55), it was 0.011, while the between group/species p-distance was 0.086. Mean pairwise tree distances within and between groups, and between groups and *H. forskali* and H. *poli* are listed in Table 3. Group 1 consisted of 12 (31.60%) of the analysed specimens (GenBank accession numbers KY774324-KY774335) and matched the nucleotide pattern of *H. mammata*. Two of the six haplotypes in Group 1 were present in more than one specimen. A maximum of 8 nucleotide differences (1.94%) was found between individuals IC-6 and LB-1. Group 2 consisted of 26 (68.40%) of the analysed specimens (GenBank accession numbers KY774336-KY774361), with 12 haplotypes recorded, of which three haplotypes were present in more than one specimen. A maximum of 8 (1.94%) nucleotide differences was found in several pairwise comparisons and they all matched the nucleotide pattern of *H. tubulosa*.

Our *H. forskali* (H. f. RO1) and *H. poli* (MD1) sequences (Genbank accession numbers KY774322 and KY774323, respectively) matched the expected species sequences from the NCBI database, suggesting the correct species assessment based on morphological characteristics (Figure 2 and Figure S1).

**Figure 3:**
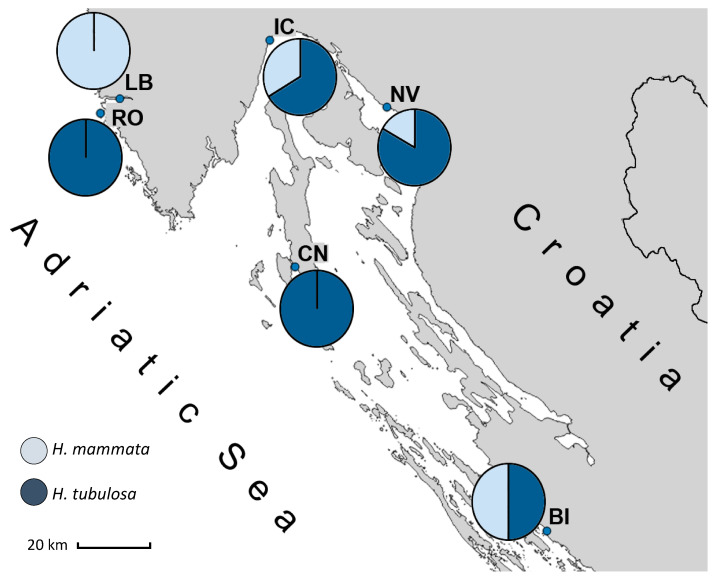
Ratio of sea cucumber specimens belonging to Group 1 (*Holothuria mammata*) and Group 2 (*Holothuria tubulosa*) at each collection site. All 6 analysed individuals at Lim Bay (LB) belonged to the species *H. mammata*, while only *H. tubulosa* was found at Rovinj (RO) and Cres–Nerezine (CN). Both species were present at Ičići (IC), Novi Vinodolski (NV), and Biograd (BI).

The specimen Z Mljet (ac. no. KY774360), previously labelled as *H.* sp. cf. *mammata* [12], was genetically grouped into Group 2, matching the *H. tubulosa* sequences (Figure 2 and Figure S1). Our results indicate that the two species (Group 1 and Group 2) are distributed throughout the Adriatic Sea, and their populations can mix. While in some locations, only one species was found, in others, both were present. Specimens collected from LB were all genetically grouped into Group 1, while the specimens from RO and CN all belonged to Group 2. The other three collection sites contained mixed populations (Figure 3).

## 4. Discussion

Until now, sea cucumber identification in the Adriatic Sea relied on exterior morphological characteristics as described by [20]. All previous research on the distribution, abundance, and biometry of *H. tubulosa* in the Adriatic Sea did not include genetic assessment and concluded that *H. mammata* is rare [12]. Stemming from this assessment, distribution and abundance censuses in the Adriatic disregarded *H. mammata*, and only *H. tubulosa*, *H. poli*, and *H. forskali* appeared in the region’s scientific literature. Genetic analysis from this research strongly points towards the presence of two species whose exterior morphologies fit the *H. tubulosa* morphospecies in the Adriatic Sea, suggesting a different species distribution, with up to a third of the *H. tubulosa* morphospecies belonging to *H. mammata.* Considering that *H. mammata* is present in relatively large numbers in the Adriatic Sea and shares its habitat with the very similar *H. tubulosa,* it is possible that the two species were previously treated as one. Thus, it is likely that previous research combined data on both species and should be used with care in the future.

Barcoding analyses showed that the mean pairwise tree distance was 7.9% between Groups, and the p-distance was more than 7 times greater between than within groups, suggesting that each group represents a species. Genetic studies of cryptic species suggest that organisms can be distinguished at the species level if the interspecific genetic distance is ten times the intraspecific distance (often 2–3%) [17,38,39]. In a large-scale barcoding study of commercially valuable holothurians from the Atlantic, Pacific, and Indian Oceans, [22] showed that conspecific variation never exceeded 4.5% and was generally <2%, while sequence differences greater than 5% almost always indicated different species.

Sequence comparison with the NCBI database reveals that Group 1 sequences closely resemble *H. mammata,* and Group 2 sequences best match *H. tubulosa.* Genetic barcoding showed that Zavodnik’s specimen, labelled as *H.* sp. cf. *mammata* in the Ruđer Bošković Institute collection in Rovinj, is indeed misidentified and is a mammilate morphotype of *H. tubulosa*, as [16] had predicted (Figure 2). Even though we did not find any research showing hybridisation between *H. mammata* and *H. tubulosa*, recent studies indicated that it is possible between *H. tubulosa* and *H. poli* [40]. Given that this analysis was carried out based on mtDNA, further analyses that combine nDNA and mtDNA markers are needed to ascertain the presence of hybrids. Nevertheless, due to 30% of our samples being genetically classified as *H. mammata*, even if hybrids were present, it is still a very strong indication that this species is present in the Adriatic Sea.

Even though some locations had only *H. mammata* or *H. tubulosa*, in other areas, both species were present, despite the small sample size (N = 6–7) collected from each location (Figure 3). It should be underlined that due to this small sample size, the proportions of each species within local holothurian populations are not representative and should be further investigated; however, these findings are still worth mentioning as they indicate that the two species may share the same habitat.

Unlike the descriptions of *H. mammata*’s preference for deeper depths, as given by the IUCN Redlist and Turk’s guide to species identification in the Mediterranean Sea [41], we found this species at depths ranging from 3 to 20 m. We did not find *H. mammata* at our only deep location (RO, 30 m). This may be due to the small sample size or local environmental conditions and does not preclude the existence of *H. mammata* in deeper habitats in the Adriatic Sea.

The result that *H. mammata* may be widely distributed in the Adriatic Sea is not unexpected. Holothurians have a relatively long-lasting planktonic phase and high larval dispersal rates [30,42]. Because the Adriatic is connected to the Mediterranean, with currents flowing northward from the Ionian Sea [31,43], the presence of species in both bodies of water, especially eurytopic species, is consistent with oceanographic expectations.

A recent checklist of the echinoderm fauna in the Adriatic Sea, based on a detailed investigation of all available literature with temporal coverage from the end of the 18th century to present-day records, has confirmed the presence of both *H. mammata* and *H. tubolosa* species [44]. Additionally, a species from the same Holothuriidae family, *H. stellati*, was added to this checklist by compiling previous records. It is known that this species has frequently been misidentified as *H. tubulosa*. This points out that, without genetic identification supported by morphological observations, the presence and distribution of certain Holothuroiidae in the Adriatic Sea remains unclear.

## 5. Conclusions

The primary aim of this study was to employ barcoding identification to identify, determine, and clarify the presence/absence of *H. mammata* in the Adriatic Sea. Ultimately, we found that *H. mammata* is relatively abundant and widespread in the Adriatic Sea, and it shares its habitat with the very similar species *H. tubulosa,* so it is possible that these two species have been previously treated as one [14,42]. It appears that in the case of heavy commercial fishing, given their lack of strict depth preferences, both species would be similarly vulnerable to exploitation threats as both have been found at depths ranging from 1 to 30 m. Thus, further research, clarification, and quantification of the presence of sea cucumber species in the Adriatic Sea is needed, so that if commercial fishing is reopened, quotas for each species can be defined.

## Figures and Tables

**Table 1:** Collection site ID and basic information on each location where sea cucumbers were sampled.

Site ID	Collection Site	GPS Position	Depth (m)	Number of Specimens	Bottom Type
LB	Lim Bay	45°08′10.8″ N 13°42′32.0″ E	12–17	6	Gravel with rocky outcroppings
RO	Rovinj	45°04′34.9″ N 13°35′53.7″ E	30	7	Sand/mud
IC	Ičići	45°18′32.7″ N 14°17′12.6″ E	4–6	6	Gravel with rocky outcroppings
CN	Cres–Nerezine	44°40′28.3″ N 14°24′38.6″ E	2–3	6	Sand/mud and Posidonia
NV	Novi Vinodolski	45°08′00.6″ N 14°45′09.7″ E	5–7	6	Gravel
BI	Biograd na moru	43°55′50.5″ N 15°26′31.2″ E	2–3	6	Sand/mud and Posidonia

**Table 2:** Haplotype name, number of sequences comprised in that haplotype, countries where the haplotype was found, and sequence names.

Haplotype Name	Nr. of Sequences Comprised	Countries Found	Sequence Names
Hap1	18	Spain, Portugal, Croatia	NV3, ZM1, RO2, RO1, IC2, IC1, CN5, CN2, BI6, BI5, BI4; H. t. Portugal 1–3; H. t. Spain 1–4
Hap2	4	Spain, Croatia	RO4; H. t. Spain 14, 17, 19
Hap3	7	Spain, Croatia	RO3, NV5, CN6, CN4; H. t. Spain 26–28
Hap4	2	Spain, Croatia	NV4, H. t. Spain 32
Hap5	2	Spain, Croatia	NV2, H. t. Spain 10
Hap6	3	Spain, Croatia	IC5, IC3, H. t. Spain 24
Hap7	10	Spain, Croatia	NV1, LB6, LB4, BI2, BI1; H. m. Spain 24, 38, 40–42
Hap8	10	Spain, Portugal, Croatia	LB5, LB2, BI3, O. d. Portugal; H. m. Portugal 2–4; H. m. Spain 22, 32, 39
Hap9	2	Spain, Croatia	LB3, H. m. Spain 47
Hap10	2	Spain, Croatia	IC4, H. m. Spain 8
Hap11	2	Spain, Croatia	MD1, H. p. Spain 2
Hap12	3	Spain	H. t. Spain 5, 8, 13
Hap13	2	Spain, Portugal	H. m. Portugal 1, H. m. Spain 6
Hap14	4	Spain	H. m. Spain 10, 14, 15, 17
Hap15	2	Spain, Mexico	H. t. Mexico, H. m. Spain 34
Hap16	3	Spain	H. a. Spain 2, 5, 6
Hap17	2	Spain	H. m. Spain 45, 51
Hap18	5	Spain	H. a. Spain 10, 13, 17, 18, 22
Hap19	3	Mexico	H. k. Mexico 2–4

**Table 3:** Mean pairwise tree distances within and between groups. N denotes the number of specimens analysed in each group.

Groups	Mean Pairwise Tree Distance	N
Mean pairwise tree distances within groups		
Group 1/*H. mammata*	0.006	12
Group 2/*H. tubulosa*	0.007	26
*H. poli*		1
*H. forskali*		1
Mean pairwise tree distances between groups		
Group 1–Group 2	0.079	
Group 1—*H. poli*	0.144	
Group 1—*H. forskali*	0.261	
Group 2—*H. poli*	0.159	
Group 2—*H. forskali*	0.275	

## Data Availability

Data are contained within the article or in the online NCBI GenBank database under accession numbers KY774322-KY774361.

## Supplementary Materials

The following supporting information can be downloaded at: https://www.mdpi.com/article/10.3390/genes14112059/s1, Figure S1: Fully expanded phylogenetic tree (maximum likelihood), unrooted, based on 129 haplotypes from partial sequences of *COI* gene obtained from this study and NCBI GenBank; Table S1: Sequence Name/Tag used, NCBI GenBank accession numbers of all the sequences used in the analysis, and Haplotype name (if more than one sequence collapsed in one identical haplotype).
