# Supplementary figures and images for: Barcoding (COI) Sea Cucumber Holothuria mammata Distribution Analysis: Adriatic Rare or Common Species?
Source: Genes (Basel). 2023 Nov 9;14(11):2059. doi: 10.3390/genes14112059 (PMC10671717; doi:10.3390/genes14112059)

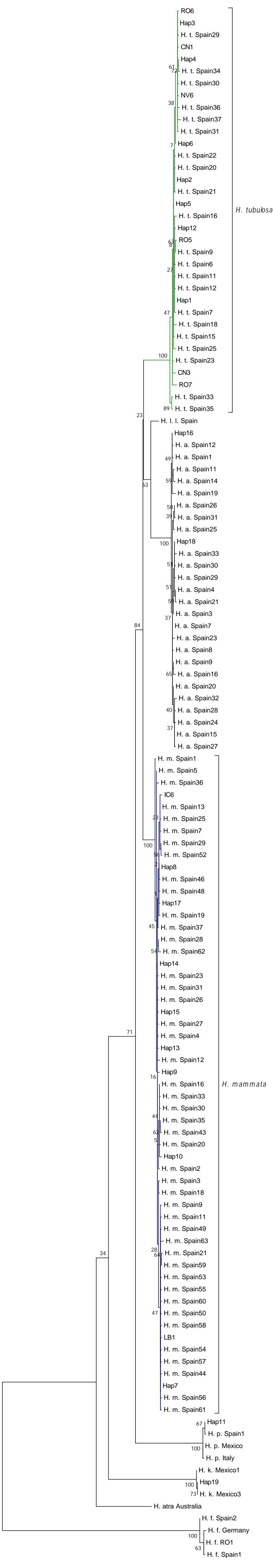

Supplement: Supplementary file 1 [file genes-14-02059-s001.zip › FigS1.pdf]
